# Supplementary material for: Chromothripsis during telomere crisis is independent of NHEJ, and consistent with a replicative origin
Source: Genome Res. 2019 May;29(5):737–49. doi: 10.1101/gr.240705.118 (PMC6499312; doi:10.1101/gr.240705.118)
Supplement: Supplemental Material [file supp_gr.240705.118_Supplemental_file_1.zip › contigs/annotated_contigs/DB114/contig.2.DB114_length_234_mean_cov_1.92307692308.docx]

**DB114_length_234_mean_cov_1.92307692308**

```
AAACTACTTTAAAGTTCATATGGAACCAAAAAAGAGCCTGCATTTCCAAGTCAATCCTAAGCCAAAAGAACAAAGCTGGAGGCTTTGTT
 >chr9:76756278-76756448 + E=2e-91
CCTGACTTCAAACTATACTACAGGCTTTGTTCCTGACTTCAAACTACCGACTTCAAACTATACTACAAGGCTATGGTAACC|CTATAGT

CT|ACCCTGAAGATTCAGACAAGCTAGATAAGAGTGAAAGATACTAACTACGAAGTTT   >chr9:79554810-79554865 - E=7e-22
```
